# Continuous care based on hierarchical nursing for cirrhosis rehabilitation: a retrospective cohort study

**DOI:** 10.3389/fmed.2026.1805360

**Published:** 2026-07-29

**Authors:** Yejing Lei, Xiaping Liu, Chenchen Zhou, Xiaoqu Zhu, Ruimin Dong, Jun Lin

**Affiliations:** Department of Infectious Hepatology, Wenzhou Hospital of TCM Affiliated to Zhejiang Chinese Medical University, Wenzhou, Zhejiang, China

**Keywords:** continuous care, hierarchical nursing, liver cirrhosis, quality of life, rehabilitation

## Abstract

**Objective:**

Cirrhosis is a progressive chronic liver disease characterized by extensive hepatic fibrosis, portal hypertension, and liver failure. This study aimed to evaluate the associations of continuous care based on hierarchical nursing with the rehabilitation and quality of life of patients with liver cirrhosis.

**Methods:**

In this retrospective cohort study, we reviewed the records of 115 patients with liver cirrhosis. Patients who received routine care between August 2022 and July 2023 formed the control group (*n* = 56), while those who were exposed to continuous care based on hierarchical nursing (implemented as part of standard practice) between August 2023 and July 2024 formed the study group (*n* = 59).

**Results:**

At 6 and 12 months, both groups showed significant decreases in Model for End-Stage Liver Disease (MELD), Liver Frailty Index (LFI), aspartate aminotransferase (AST), alanine aminotransferase (ALT), Self-Rating Anxiety Scale (SAS), and Self-Rating Depression Scale (SDS) from baseline (*p* < 0.05), with the study group demonstrating lower values than the control group (*p* < 0.05). Self-management scores, 36-Item Short Form Health Survey (SF-36) scores, and nursing quality increased significantly in both groups over time (*p* < 0.05), with the study group achieving higher scores (*p* < 0.05). The study group also exhibited a greater improvement in Child-Pugh grade distribution, a higher nursing satisfaction rate (91.53% vs. 71.43%, *p* < 0.05), and a lower complication rate (8.47% vs. 25.00%, *p* < 0.05).

**Conclusion:**

In this retrospective analysis, exposure to continuous care based on hierarchical nursing was associated with superior outcomes compared to routine care, including enhanced quality of life, liver function, self-management, and reduced complications, warranting further prospective validation.

## Introduction

Cirrhosis is a progressive chronic liver disease characterized by extensive hepatic fibrosis, portal hypertension and liver failure (1). The development of complications of liver dysfunction (ascites, hepatic encephalopathy and variceal bleeding, etc.) marks the transition from an asymptomatic phase of cirrhosis (compensated cirrhosis) to a rapidly progressive phase (decompensated cirrhosis) (1). Cirrhosis is characterised by a high mortality rate and unpredictable illness trajectories (2). Patients with this condition often experience significant physical and mental burdens. The majority report multiple distressing symptoms, including muscle spasms, sleep disturbance, chronic pain, and depression (3), which severely compromise their quality of life. Some surveys have shown that cirrhosis was associated with 2.4% of deaths globally in 2019, making it already one of the top 12 causes of death globally (4), and the cirrhosis-associated population was showing an expanding trend in the coming decade (5).

A study indicates that active lifestyle intervention toward patients with cirrhosis may help delay cirrhosis from the compensated to the decompensated state, and lifestyle intervention combined with personalized care had also shown positive effects in patients with decompensated cirrhosis (6). Although much of the cirrhosis process is either delayed or improved with personalized care, cirrhosis nursing — defined as the comprehensive, long-term nursing management specifically tailored to the physical, psychological, and social needs of patients with cirrhosis — has received less attention than other chronic diseases (7). Hepatitis, liver fibrosis, cirrhosis and liver cancer are the different stages of development of chronic liver disease, and once they have progressed to the cirrhosis stage they are very difficult to reverse, and patients with cirrhosis usually require long-term care or even lifelong care (8). Investigations have found that the clinical care model for patients with cirrhosis usually revolves around liver specialists, which makes it difficult to provide comprehensive, high-quality and personalised care management for patients due to time factors (9). Studies have found that continuity of care and nurse hierarchical management model (NHMM) have shown benefits in chronic disease management (10–12).

Continuity of care (COC), as a patient-centered, systematic model, usually refers to a series of actions designed to ensure that patients receive collaborative and continuous care across different healthcare settings (e.g., from hospital to home) and over time (10). It has shown positive results in the management of various chronic diseases (13, 14), aiming to bridge the gaps between different healthcare services and prevent fragmented care. Previous studies on cirrhosis care have demonstrated that many cirrhotic patients tend to be irritable or even resistant to treatment (3, 15). However, maintaining continuity of care can effectively mitigate these issues, reduce the incidence of complications, and enhance the quality of life for patients with cirrhosis.

As an emerging management concept, NHMM organizes and allocates nursing human resources scientifically based on the competencies, qualifications, and clinical expertise of nurses, establishing clear positional roles and responsibilities for various levels (12). This model optimizes the utilization of nursing resources by ensuring that complex patients are cared for by the most appropriate and skilled nursing staff, and has been shown to yield positive outcomes in the treatment and rehabilitation of both acute and chronic conditions (12). In addition, some studies have reported that the nurse hierarchical management model combined with continuity of care contributes to the quality of care (11). However, studies on the effectiveness of the nurse hierarchical management model combined with continuity of care for patients with cirrhosis are lacking.

Based on literature review and clinical practice evolution, our hospital implemented continuous care based on hierarchical nursing as a standard nursing pathway for cirrhosis patients in August 2023, superseding the prior routine care model. Therefore, the aim of this study was to retrospectively analyze and compare the outcomes of cirrhosis patients under these two sequentially adopted care models during the in-hospital and post-discharge phases, so as to provide preliminary real-world evidence and a practical reference for optimizing nursing strategies in patients with liver cirrhosis.

## Materials and methods

### General information

This study is a retrospective, single-center investigation. We analyzed data from patients with liver cirrhosis admitted to Wenzhou Hospital of TCM Affiliated to Zhejiang Chinese Medical University (a Grade A tertiary hospital in Wenzhou, China) between August 2022 and July 2024.

In August 2023, our hospital adopted a revised standard nursing protocol, integrating a two-component model consisting of (1) a three-tiered nurse hierarchical management system (primary, senior, and charge nurses) and (2) a post-discharge continuity of care program (regular telephone follow-ups, personalized care plans, and a 24-h hotline) into the conventional treatment framework for cirrhosis patients. This model is hereafter referred to as “continuous care based on hierarchical nursing.” In this study, patients admitted during the period of conventional treatment (August 2022–July 2023) were classified as the control group, while those admitted under the new integrated care model (August 2023–July 2024) were classified as the study group.

Initially, 130 patients underwent eligibility assessment. Among them, 11 did not meet inclusion criteria, and 4 were excluded due to incomplete medical records. Ultimately, 115 patients participated in the study: 56 in the control group and 59 in the study group.

The study was approved by the Ethics Committee of Wenzhou Hospital of TCM Affiliated to Zhejiang Chinese Medical University (Ethics Approval Number: WZY2024-LW-071-01).

### Sample size and *post-hoc* power analysis

Given the retrospective design of this study, a prospective sample size calculation was not performed. However, a *post-hoc* power analysis was conducted to assess whether the enrolled sample size provided adequate statistical power to detect the observed between-group difference in the primary outcome (MELD score at 12 months). The analysis was performed using PASS software (version 15) with the “Tests for Two Means (Simulation)” module. Based on the observed data (Control group: mean = 10.52, SD = 1.55, *n* = 56; Study group: mean = 9.14, SD = 1.36, *n* = 59), and setting a two-sided alpha level of 0.05 with 10,000 Monte Carlo simulations, the calculated statistical power was 99.89%. This far exceeds the conventional threshold of 80%, confirming that the study had ample power to detect the clinically significant difference in MELD scores between the two groups.

### Inclusion and exclusion criteria

Inclusion criteria: patients diagnosed with cirrhosis through liver biopsy, elastography, ultrasound or CT scan (16); patients with stable vital signs (i.e., no hemodynamic instability or need for vasopressors) and no severe concurrent systemic diseases; age less than 75 years at the time of admission; patients with a Child-Pugh grade of A-C receiving treatment at our hospital.

Exclusion criteria: patients requiring immediate liver transplantation; patients with active severe drinking habits (i.e., ongoing alcohol dependence or repeated withdrawal episodes within recent months) — those with past alcohol dependence but sustained abstinence prior to admission were not excluded; patients with acute hepatic encephalopathy (Grade ≥2 per West Haven criteria) at admission (those with well-controlled history without acute decompensation were not automatically excluded); patients with severe non-liver disease and impaired short-term prognosis (e.g., severe or uncontrolled cardiovascular, respiratory, renal, endocrine, or autoimmune diseases); patients with hepatocellular carcinoma or other non-liver malignancies; patients with acute liver failure; pregnant or lactating patients; patients with documented mental or cognitive impairments.

### Nursing procedure

**Figure 1:**
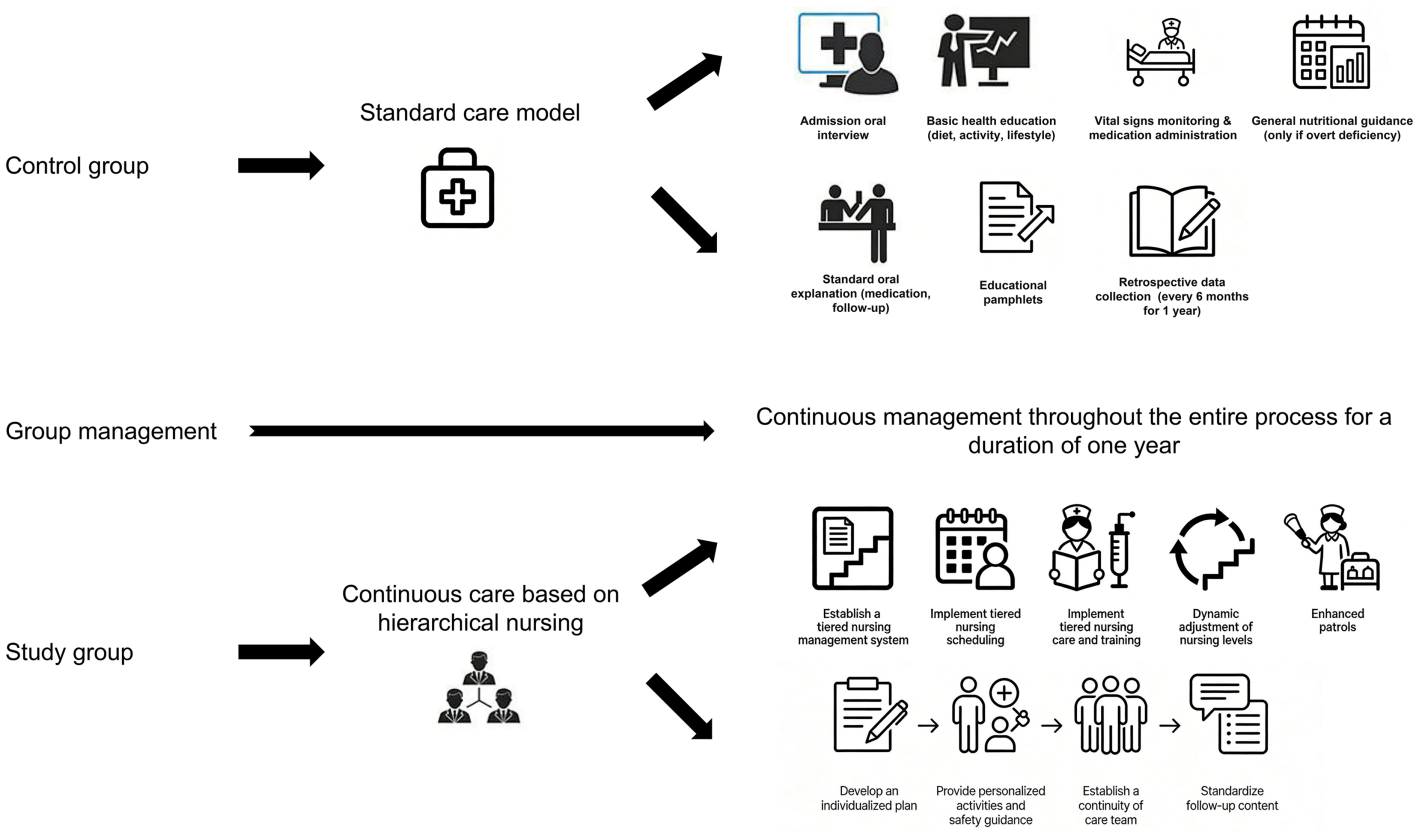
Schematic diagram of intervention for two patient groups.

The control group received routine care (routine post-discharge follow-up, general dietary advice, basic discharge education by general ward nurses), whereas the study group received continuous care based on hierarchical nursing (structured telephone follow-ups with a 24-h hotline, personalised meal plans, a three-tiered nursing team, and an individualised continuing care plan). Full details are provided in Figure 1 and the following subsections.

Control group (routine nursing mode, *n* = 56). Patients received conventional care based on components described previously (17). Upon admission, they underwent a formal oral interview and received basic health education (lifestyle adjustments, low-sodium moderate-protein diet, appropriate physical activity). During hospitalization, routine nursing support included vital signs monitoring, medication administration, and general nutritional guidance per standard hospital protocols; nutritional supplementation was given only when overt deficiency was documented. At discharge, patients were provided with standard oral explanations and educational pamphlets covering medication schedules, follow-up arrangements, and reinforced lifestyle/dietary recommendations. Routine post-discharge follow-up (e.g., via WeChat) was conducted, and patient data (readmissions, complications, overall health status) were collected retrospectively from medical records every 6 months over 1 year.

Study group (continuous care based on hierarchical nursing, *n* = 59). This group received the hospital’s integrated protocol, which comprised three interlinked components: a three-tiered nursing system, structured in-hospital stratified care, and a post-discharge continuity of care program. All key elements are summarized here; detailed measures are retained in the original description.

(1) Three-tiered nursing system: A formal hierarchical system was established (12), categorizing nurses into primary (>2 years hepatology experience), senior (>5 years), and charge nurses (>10 years with leadership roles). All completed mandatory, tier-specific training and certification. Nursing shifts were organized into fixed teams that combined different levels to ensure skill integration: basic care teams (primary and senior), complex care teams (primary and charge), and emergency/critical care teams (primary, senior and charge).
(2) Stratified in-hospital care: Upon admission, primary nurses monitored vital signs, and charge nurses conducted a comprehensive assessment of the patient’s condition and self-care ability using a standardized form for rapid triage. Patients with mild conditions and complete self-care ability received basic nursing and health education from primary-senior teams. Those with stable conditions but partial self-care dependence received more frequent inspections and reinforced care (including charge nurse involvement). Patients with severe conditions were regularly monitored by teams including senior and charge nurses. In emergencies, a charge nurse-led team performed first aid per a predefined protocol. Nurses assisted physicians in regularly adjusting nursing levels based on patient status. Primary nurses conducted hourly ward rounds, and senior/charge nurses evaluated nursing effects at least daily (12). Intensive, personalized nutritional management was a core component: a senior nurse performed a comprehensive initial assessment, developed an individualized meal plan tailored to liver function and nutritional status, provided guidance on optimal meal frequency/composition to combat muscle wasting, continuously monitored and adjusted the plan during follow-ups, and actively involved family caregivers to ensure home adherence. This enhanced nutritional regimen was provided in addition to the hospital’s standard nutritional protocols (unchanged throughout the study period), as an added component of the hierarchical nursing intervention.
(3) Post-discharge continuity of care: Prior to discharge, the responsible nurse developed a personalized continuing care plan for each patient, addressing medication adherence, dietary management, symptom monitoring, and regular follow-up scheduling. Personalized physical activity guidance and safety counseling were provided (tailored, home-based, based on LFI score and functional status). A continuing care team (patient‘s assigned senior or charge nurse) conducted structured telephone follow-ups: weekly for the first month post-discharge, bi-weekly for months 2–3, and monthly thereafter until 12 months (18). Each follow-up used a standardized checklist (medication review, dietary log, symptom screening for ascites/hepatic encephalopathy, self-management progress) and lasted 15–20 min. A clear missed-call protocol and an emergency escalation pathway (including immediate notification of the on-call hepatologist) were in place. A 24-h care hotline was offered for urgent inquiries (18).

### Data collection and sources

All data for this retrospective analysis were extracted from the hospital’s electronic medical records (EMR) system, archived nursing documentation, and completed patient-reported outcome questionnaires stored in patient files. A standardized data extraction form was used by two independent researchers to ensure accuracy. Discrepancies were resolved by consensus or consultation with a third senior investigator. Baseline and follow-up data at 6 and 12 months were collected for all outcome measures.

We reviewed institutional protocol records (Pharmacy and Therapeutics Committee, Medical Quality Management Office) for 2022–2024 and confirmed no changes in antiviral formularies/treatment algorithms, diuretic/beta-blocker dosing, nutritional supplement formularies, or MELD/Child-Pugh diagnostic criteria. These records are audited annually.

### Assessment criteria

The primary outcomes focused on objective measures of liver function and prognosis, including the Model for End-Stage Liver Disease (MELD) score (19), the Liver Frailty Index (LFI) for assessing frailty (20), and Child-Pugh grade. Key liver function biochemical markers, including serum aspartate aminotransferase (AST) and alanine aminotransferase (ALT), were measured using standard enzymatic photometric methods on an automated biochemical analyzer. Safety was assessed by monitoring the incidence of major complications (ascites, hepatic encephalopathy, variceal bleeding, and renal failure) over the entire one-year study period, with the incidence rate calculated as (number of complications / total cases) × 100%. All primary outcomes had been assessed at baseline, 6 months, and 12 months as part of standard clinical follow-up.

Secondary outcomes encompassed patient-reported and functional measures, including quality of life evaluated using the SF-36 questionnaire (21) (covering physical function, bodily pain, and mental health, among eight domains), self-management abilities measured by the Self-Management Scale (SMS) (22), psychological status assessed via the Self-Rating Anxiety Scale (SAS) (23) and Self-Rating Depression Scale (SDS) (24), and patient satisfaction with nursing care using a structured satisfaction questionnaire (12). Nursing quality was also evaluated as a secondary outcome at 6 and 12 months using a specialized survey (25).

General patient characteristics—including gender, age, BMI, MELD score, LFI, Child-Pugh score, etiology of cirrhosis—were recorded at baseline for group comparison. All data for the above outcomes are obtainable through a combination of retrospective medical record extraction assessment.

### Statistical analysis

Data were analyzed using SPSS 26.0 software. Normally distributed measurements are expressed as mean ± standard deviation (SD). For longitudinal data (e.g., MELD, LFI), linear mixed models were applied to analyze group, time, and interaction effects, with Bonferroni-corrected simple-effects tests for post-hoc comparisons (reported with effect sizes and 95% CIs). For single time-point comparisons, independent-samples or paired *t*-tests were used as appropriate. Skewed data are expressed as median (IQR) and compared using Mann–Whitney U (between groups) or Wilcoxon tests (within group). Categorical data are presented as *n* (%) and analyzed by *χ*^2^ test. A two-sided *p* < 0.05 was considered statistically significant.

## Results

### Baseline characteristics were comparable between groups

Table 1 summarises the baseline clinical characteristics of the 115 enrolled participants, all of which were collected at the time of hospital admission (in-patient). No significant differences were observed between the control and study groups in participant characteristics (*p* > 0.05). These findings indicate that the two groups were comparable at baseline.

**Table 1:** Baseline characteristics of patients from the control group and the study group.

General clinical data		Control group (*n* = 56)	Study group (*n* = 59)	t/Z/χ^2^	*p*
Gender [*n* (%)]	Male	38 (67.86)	35 (59.32)	0.903	0.342
	Female	18 (32.14)	24 (40.68)		
Age (years, *x̄* ± s)		62.32 ± 7.94	59.66 ± 8.69	1.704	0.091
BMI (kg/m^2^, *x̄* ± s)		23.65 ± 3.44	22.56 ± 3.86	1.595	0.114
MELD score (points, *x̄* ± s)		11.98 ± 2.01	11.34 ± 2.03	1.70	0.092
LFI (points, *x̄* ± s)		3.99 ± 0.45	3.96 ± 0.43	0.315	0.753
Child-Pugh score [M (IQR)]		7.30 (2.50)	6.90 (1.30)	−0.118	0.906
Ascites [*n* (%)]	None	22 (39.29)	26 (44.07)	0.395	0.821
	Mild	20 (35.71)	18 (30.15)		
	Moderate–Severe	14 (25.00)	15 (25.42)		
History of hepatic encephalopathy [*n* (%)]		9 (16.07)	12 (20.34)	0.207	0.649
Esophageal varices	None	18 (32.14)	21 (35.59)	0.210	0.900
	Mild	25 (44.64)	24 (40.68)		
	Severe	13 (23.21)	14 (23.73)		
Aetiology [*n* (%)]	ALD	44 (78.57)	48 (81.36)	0.993	0.963
	HBV	3 (5.36)	2 (3.39)		
	HCV	2 (3.57)	2 (3.39)		
	MALSD	8 (14.29)	6 (10.17)		
	PBC	1 (1.79)	1 (1.69)		
	others	2 (3.57)	1 (1.69)		
Pre-admission alcohol consumption (g ethanol/day, *x̄* ± s)		68.42 ± 11.23	71.2 ± 12.15	1.272	0.206
Duration of abstinence prior to admission (months, *x̄* ± s)		2.1 ± 0.51	2.0 ± 0.43	1.139	0.257

M, male; F, female; ALD, Alcohol related liver disease; HBV, hepatitis B virus; HCV, hepatitis C virus; MALSD, metabolic dysfunction related steatosis liver disease; PBC, primary biliary cirrhosis.

### Primary outcome

#### MELD and LFI scores decreased over time, with lower scores in the study group

**Figure 2:**
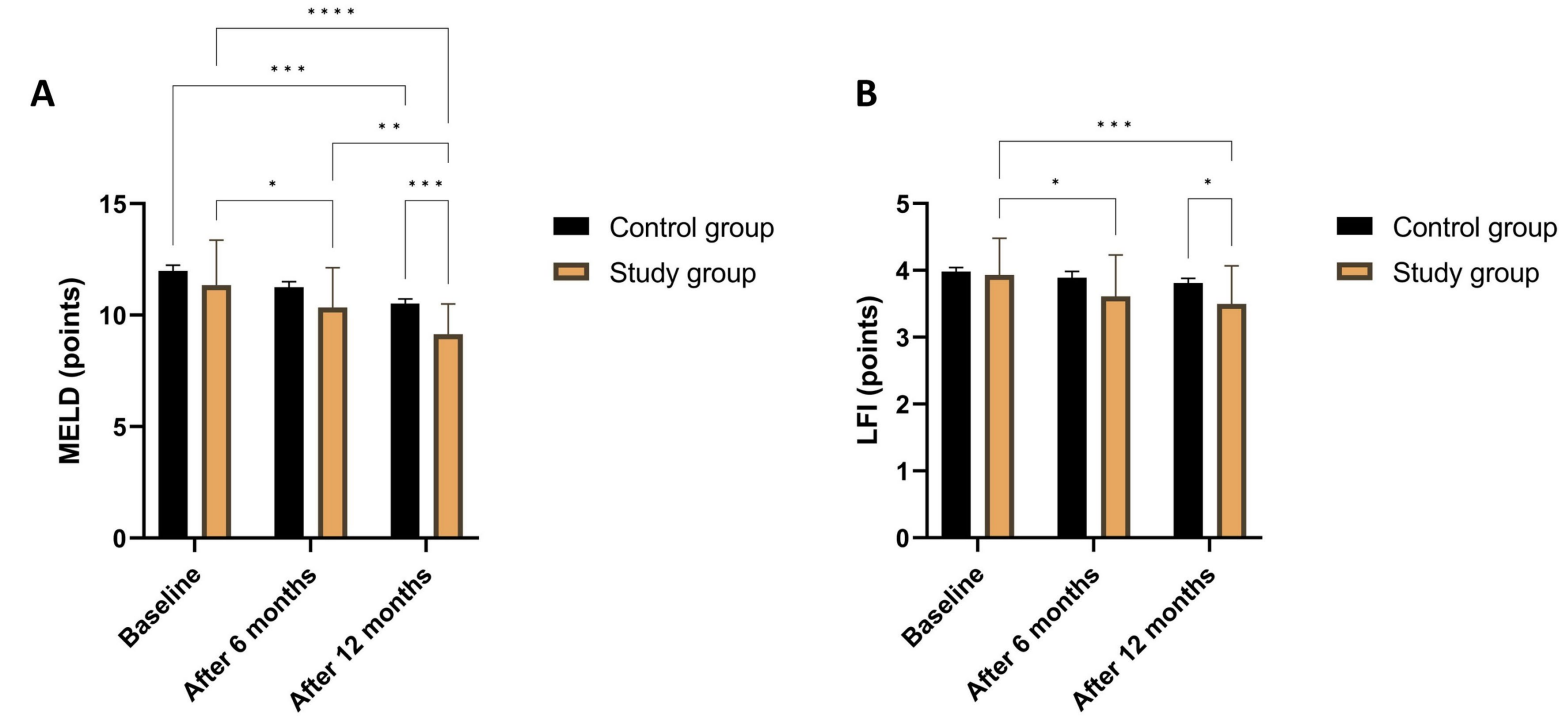
Comparison of the LFI and MELD scores. **(A)** LFI scores. **(B)** MELD scores. ^*^p < 0.05, ^**^p < 0.01, ^***^p < 0.001, ^****^p < 0.0001; LFI, liver frailty index; MELD, model for end-stage liver disease.

There were no significant differences in baseline LFI and MELD scores between the two groups (*p* > 0.05). Compared with baseline levels, MELD scores and LFI gradually decreased at the 6-month and 12-month follow-ups in both groups, with the study group showing lower scores than the control group. See Figure 2.

#### AST and ALT levels decreased in both groups, with greater reductions in the study group

**Figure 3:**
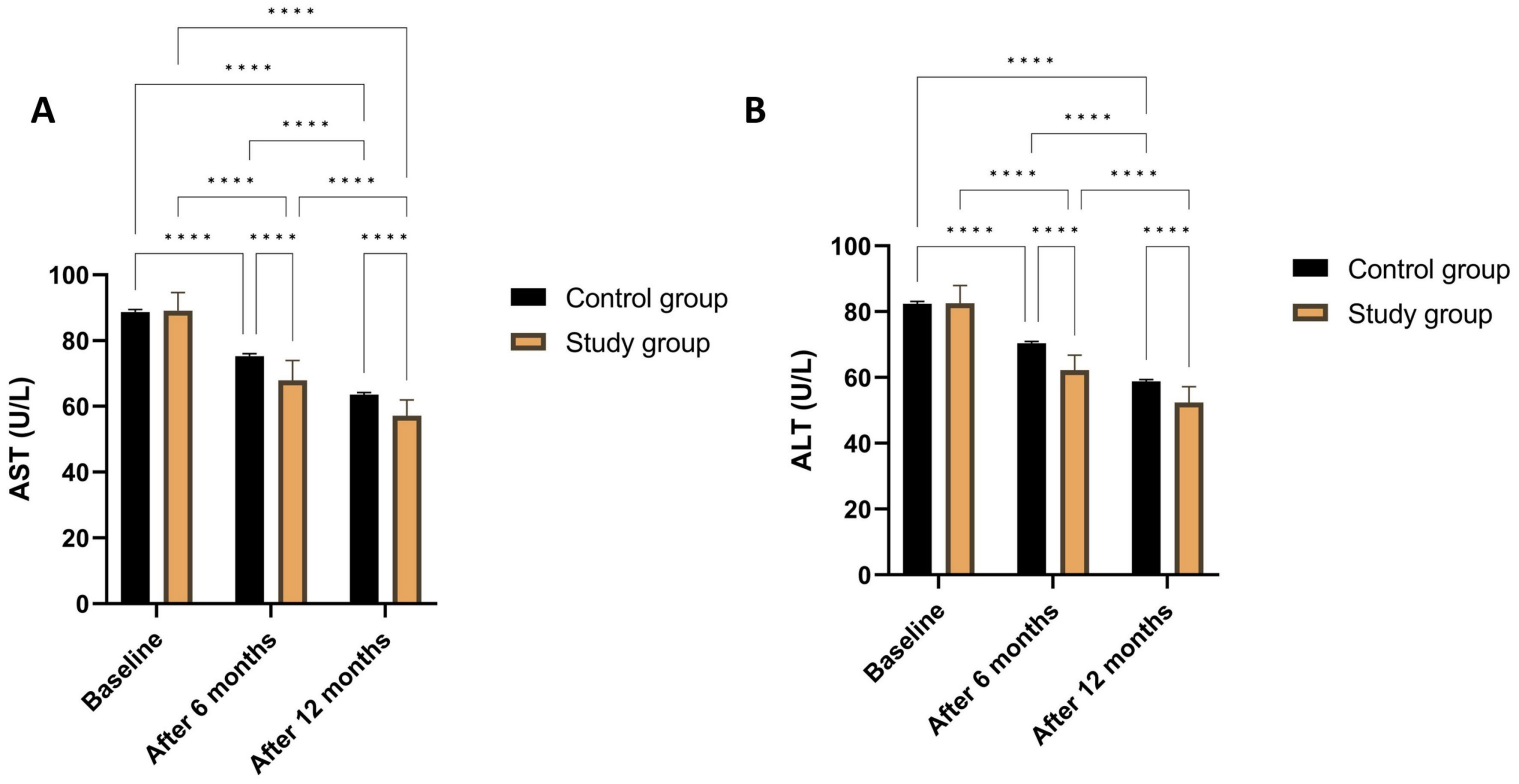
Comparison of the liver function indicators. **(A)** AST levels. **(B)** ALT levels. ^****^p < 0.0001; AST, Aspartate aminotransferase; ALT, Alanine aminotransferase.

There were no significant differences in baseline AST and ALT levels between the two groups (*p* > 0.05). Compared with baseline levels, both groups showed a gradual decrease in AST and ALT at the 6-month and 12-month follow-ups (*p* < 0.05), with the study group exhibiting significantly lower values than the control group (*p* < 0.05). See Figure 3.

Individual synthetic function parameters (albumin, total bilirubin, and INR) at baseline, 6 months, and 12 months are presented in Table 2. No significant differences were observed between the two groups at baseline (all *p* > 0.05). At 6 and 12 months, the study group showed significantly higher albumin levels and significantly lower total bilirubin and INR values compared to the control group (all *p* < 0.05), consistent with the improvements observed in the composite MELD and Child-Pugh scores.

**Table 2:** Individual synthetic function parameters at baseline, 6 months, and 12 months.

Parameter	Time point	Control group (*n* = 56)	Study group (*n* = 59)	*p*
ALB (g/L)	Baseline	32.84 ± 5.12	33.01 ± 5.48	0.864
	6 months	33.92 ± 4.56	36.27 ± 4.39	0.006
	12 months	34.15 ± 4.73	38.84 ± 4.02	<0.001
TBIL (μmol/L)	Baseline	28.36 ± 12.05	27.92 ± 11.74	0.843
	6 months	25.71 ± 10.38	20.54 ± 8.82	0.005
	12 months	24.88 ± 9.75	16.38 ± 6.91	<0.001
INR	Baseline	1.38 ± 0.32	1.37 ± 0.30	0.863
	6 months	1.34 ± 0.28	1.24 ± 0.22	0.035
	12 months	1.32 ± 0.26	1.16 ± 0.18	<0.001

Data are presented as mean ± SD. *p* values represent between-group comparisons at each time point (linear mixed model or independent *t*-test). ALB, albumin; TBIL, total bilirubin; INR, international normalized ratio.

#### Child-Pugh grade distribution improved, with a greater shift toward grades a/B in the study group

During the nursing process, both groups showed an increasing trend in the number of patients with Grade A and B liver function, while the number of patients with Grade C liver function decreased. The trend of change in the study group was greater than that in the control group. See Table 3.

**Table 3:** Comparison of the Child-Pugh grade [*n* (%)].

Period	Grade	Control group (*n* = 56)	Study group (*n* = 59)	χ^2^	*p*
Baseline	A	9 (16.07)	10 (16.95)	0.048	0.976
	B	26 (46.43)	28 (47.46)		
	C	21 (37.50)	21 (35.59)		
After 6 months	A	12 (21.43)	14 (23.73)	0.359	0.836
	B	27 (48.21)	30 (50.85)		
	C	17 (30.36)	15 (25.42)		
After 12 months	A	15 (26.79)	18 (30.51)	1.634	0.442
	B	26 (46.40)	31 (52.54)		
	C	15 (26.79)	10 (16.95)		

#### Complication rate was lower in the study group

Following nursing intervention, the incidence of complications in the study group (8.47%) was significantly lower than that in the control group (25.00%), with a statistically significant difference (*p* < 0.05). See Table 4.

**Table 4:** Comparison of the complication rates [*n* (%)].

Group	Ascites	Hepatic encephalopathy	Bleeding from esophageal or gastric varices	Renal failure	Total complication rates
Control group	12.50 (7/56)	5.36 (3/56)	3.57 (2/56)	3.57 (2/56)	25.00 (14/56)
Study group	5.08 (3/59)	1.69 (1/59)	1.69 (1/59)	0.00 (0/59)	8.47 (5/59)
χ^2^					5.689
*p*					0.017

### Secondary outcome

#### Self-management and SF-36 scores increased over time, with higher scores in the study group

**Figure 4:**
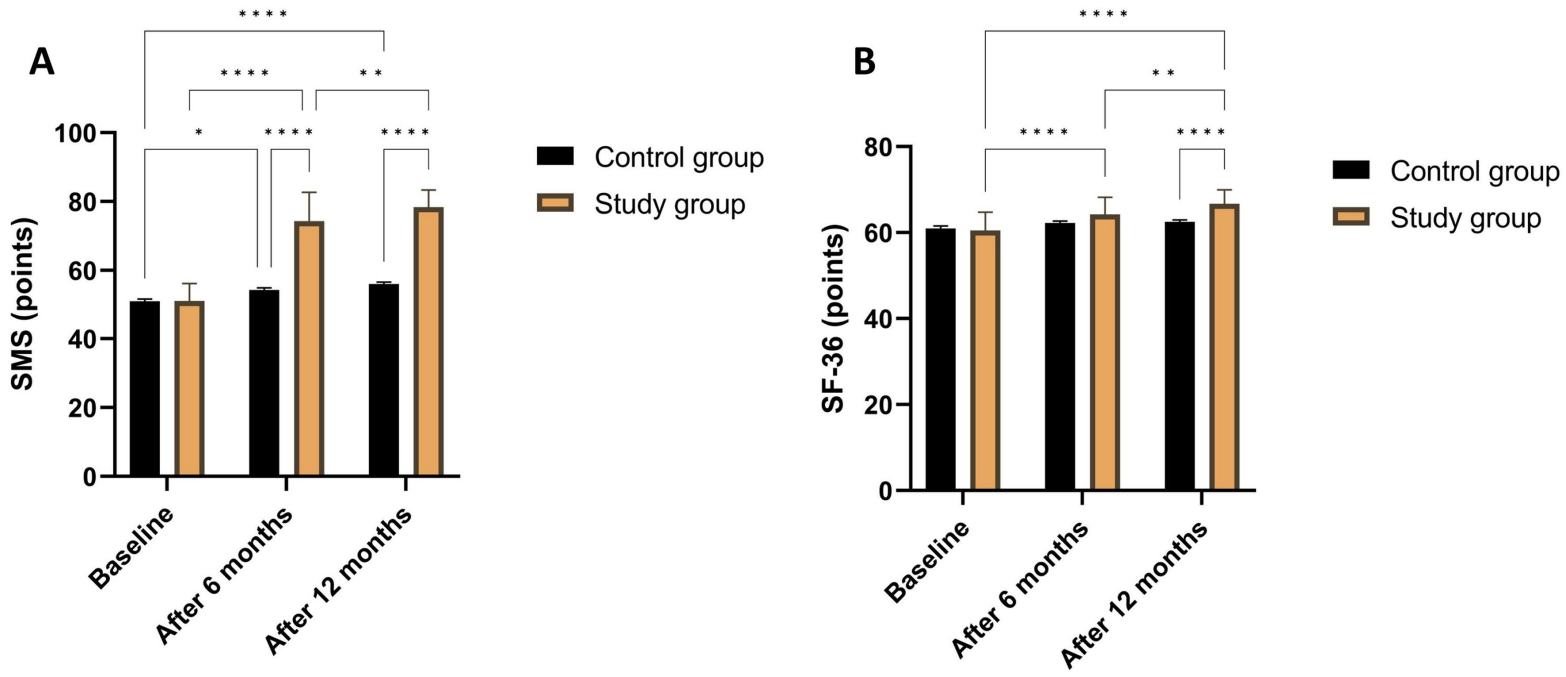
Comparison of the SF-36 and SMS (points, *x̄* ± s). **(A)** SF-36 scores. **(B)** SMS scores. ^*^p < 0.05, ^**^p < 0.01, ^****^p < 0.0001; SMS, self-management scale; SF-36, Medical Outcomes Study 36-Item Short Form Health Survey.

There were no significant differences in baseline self-management scores and SF-36 Scores between the two groups (*p* > 0.05). Compared with baseline levels, self-management scores and SF-36 scores gradually increased in both groups at the 6-month and 12-month follow-ups, with the study group showing higher scores than the control group. See Figure 4.

#### SAS and SDS scores decreased over time, with greater reductions in the study group

**Figure 5:**
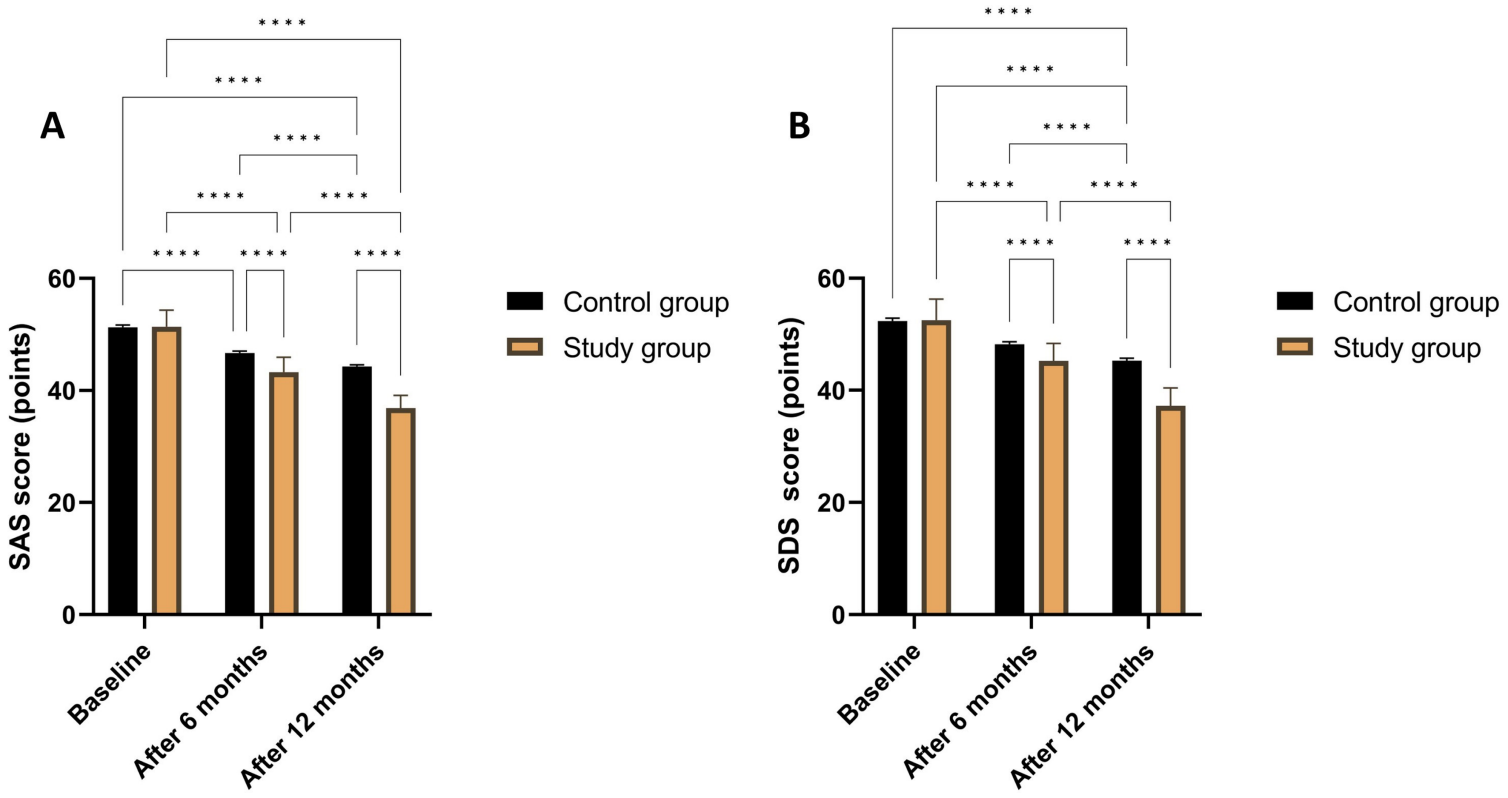
Comparison of the psychological conditions (points, *x̄* ± s). **(A)** SAS scores. **(B)** SDS scores. ^****^p < 0.0001; SAS, self-rating anxiety scale; SDS, self-rating depression scale.

Baseline SAS and SDS scores showed no significant differences between the two groups (*p* > 0.05). Compared with baseline levels, both SAS and SDS scores gradually decreased at the 6-month and 12-month follow-ups (*p* < 0.05), with the study group exhibiting significantly lower scores than the control group (*p* < 0.05). See Figure 5.

#### Nursing satisfaction was higher in the study group

At the end of the observation period, the control group’s nursing satisfaction rate (71.43%) was significantly lower than that of the study group (91.53%), with the difference being statistically significant (*p* < 0.05). See Table 5.

**Table 5:** Comparison of the nursing satisfaction [*n* (%)].

Group	Dissatisfied	Satisfied	Very satisfied	Total nursing satisfaction
Control group	16 (28.57)	35 (62.50)	5 (8.93)	40 (71.43)
Study group	5 (8.47)	20 (33.89)	34 (57.63)	54 (91.53)
χ^2^				7.774
*p*				0.005

Dissatisfied: 0–60 points; Basically satisfied: 60–84 points; Very satisfied: above 85 points; Total nursing satisfaction: Basically satisfied + Very satisfied.

#### Nursing quality scores improved over time, with higher scores in the study group

After 6 months, the nursing quality in the study group was significantly higher than that in the control group (*p* < 0.05). Compared with the 6-month assessment, the nursing quality scores of both groups increased significantly at 12 months (*p* < 0.05), with the study group showing a significantly higher score than the control group (*p* < 0.05). See Table 6.

**Table 6:** Comparison of the nursing quality (points, *x̄* ± s).

Parameters	Period	Control group	Study group	t	*p*
Nursing quality	After 6 months	65.18 ± 14.58	82.74 ± 10.35	−7.526	<0.001
	After 12 months	70.29 ± 12.38^b^	85.91 ± 6.10^b^	−8.614	<0.001

Compared with After 6 months, ^b^*p* < 0.05.

## Discussion

This retrospective cohort study aimed to evaluate the associations between continuous care based on hierarchical nursing and various patient outcomes in individuals with liver cirrhosis. Our analysis revealed that receipt of this structured nursing approach was associated with significantly better outcomes across multiple domains—including physiological indicators, psychosocial adaptation, self-management behaviors, and clinical endpoints—compared to routine care alone.

This study observed a series of clinically significant improvements in physiological function and disease severity, reflected across multiple interrelated indicators. First, the significant reduction in LFI (*p* < 0.05) suggests a potential association between the intervention and improved muscle mass and physical functional status, consistent with trends observed in previous studies (26). This improvement likely stemmed from the individualized nutritional support program and graded exercise guidance implemented in the study: seven personalized meal plans developed based on comprehensive nutritional assessments, combined with a six-meal feeding regimen. The multi-meal feeding approach not only optimized patients’ energy intake patterns but may also have promoted muscle mass maintenance and recovery by improving protein synthesis and metabolic balance (27). Furthermore, the encouragement of safe, guided physical activity likely contributed to maintaining functional mobility and mitigating muscle disuse, while avoiding the risk of elevated portal vein pressure (28). Notably, the tiered management system achieved a clinically significant average reduction of 0.4–0.5 in the LFI score. Previous studies indicate that a 0.3 reduction in LFI correlates with improved survival rates, while a 0.5 reduction demonstrates substantial benefits even for patients with advanced cirrhosis (29, 30). Second, the significant decrease in MELD scores (*p* < 0.05) reflects overall improvements in liver function and prognosis. This change likely correlates with the refined condition monitoring and timely interventions enabled by the tiered management model: a three-tiered nursing team regularly assessed patient status, assisting physicians in rapidly adjusting treatment plans; hourly rounds by primary nurses ensured early detection of clinical changes; and the continuous implementation of individualized nutritional support likely promoted hepatocyte recovery by reducing metabolic burden and inflammatory responses (18). Notably, both groups showed significant decreases in MELD scores over time, yet the study group exhibited a more pronounced and sustained downward trend compared with the control group, highlighting the added value of comprehensive nursing interventions in slowing disease progression. Regarding liver function biomarkers, the significant reduction in AST and ALT levels (*p* < 0.05) indicates diminished hepatocyte injury and improved hepatocyte membrane stability, consistent with findings reported by Huo et al. (31). This change likely resulted from the synergistic effects of multiple interventions: continuous nutritional support provided essential nutrients and antioxidants for hepatocyte repair; tiered management ensured early identification and management of potential hepatotoxic factors; and enhanced overall treatment adherence prevented behaviors that could exacerbate liver injury. This further validates the positive role of the integrated care model in mitigating hepatic inflammatory responses and promoting hepatocyte repair. Finally, the improving trend in Child-Pugh classification (increased proportion of grades A/B, decreased proportion of grade C), though not statistically significant between groups, holds important clinical reference value (32). This trend suggests that the integrated model of in-hospital hierarchical management followed by post-discharge continuity of care may improve hepatic synthetic, metabolic, and excretory functions through multiple pathways: preventive management of complications such as jaundice, ascites, and hepatic encephalopathy by tertiary care teams; individualized nutritional plans optimizing protein-energy expenditure and water-electrolyte balance; and continuous health education enhancing patient self-management capabilities. Collectively, these factors promote improved hepatic reserve function and delayed disease progression. The consistent improvement in these physiological indicators demonstrates that continuous care based on hierarchical nursing achieves effective control of disease severity in cirrhosis patients through multi-targeted, multi-pathway intervention mechanisms, laying a solid foundation for improving long-term patient outcomes.

In terms of self-management capabilities and overall nursing quality, patients in the intervention group demonstrated higher self-management scale scores and nursing satisfaction (*p* < 0.05). This outcome reflects how the tiered management model significantly enhanced the nursing team’s overall professional competence and service capacity through systematic training, clear role definition, and standardized operational procedures. Tier-3 nurses not only assume roles in clinical supervision and emergency decision-making but also conduct bedside mentoring and competency development for primary nurses. The participatory nursing approach employed in continuity of care—such as encouraging patients to record nutritional diaries and collaboratively develop daily meal plans—effectively stimulated patients’ autonomy and initiative in health management. This shifted their role from “passive treatment recipients” to “active management participants.” This “empowerment” model aligns with contemporary chronic disease management trends (33, 34), offering novel insights for improving long-term treatment adherence and patient outcomes.

In terms of improving psychological well-being and health-related quality of life, the intervention group demonstrated significantly greater improvements than the control group in SAS, SDS, and SF-36 scores (*p* < 0.05). This outcome highlights how the nursing model employed in this study actively addresses patients’ psychosocial needs while prioritizing their physical health. The integration of one-on-one guidance, structured health education courses, and continuous nurse–patient communication within the continuity of care program significantly enhanced patients’ disease awareness and self-efficacy, thereby alleviating anxiety and depression stemming from disease uncertainty and treatment burdens (15). Furthermore, the tiered management framework enhanced patients’ trust and sense of security within the healthcare environment by establishing clear nursing levels and coherent service processes. This played a crucial role in alleviating psychological stress and fostering a positive attitude toward treatment (35). These findings align with conclusions from multiple prior studies (36), further supporting the integration of psychosocial support as a core component of chronic disease management systems.

Regarding the safety of complication prevention and management, although the intergroup differences in Child-Pugh classification changes did not reach statistical significance, the significant reduction in complication incidence in the intervention group remains clinically important. This outcome may be attributed to the risk alert and rapid response processes documented within the tiered management system: Early identification of high-risk patients by senior nurses, targeted nutritional and medication adjustments, and standardized emergency protocols collectively formed an effective risk control system. Furthermore, continuity of care emphasized daily training for patients and families, enhancing their ability to recognize early symptoms of potential complications such as gastrointestinal bleeding and hepatic encephalopathy. This enabled timely and effective interventions even after discharge, preventing disease progression. It is particularly noteworthy that a significant proportion of patients in this study were in the decompensated stage. The collaborative nursing model implemented in the studied cohort demonstrated unique advantages in rational medication supervision and adverse reaction monitoring.

This study fills a gap in the existing evidence by specifically evaluating the combination of a tiered nursing management model with continuity of care in patients with liver cirrhosis. To our knowledge, no prior investigation has reported on this integrated approach in this population. However, several limitations should be acknowledged. The retrospective, time-sequential design carries a risk of temporal confounding. We verified that no cirrhosis-related clinical protocols (antiviral, diuretic/beta-blocker, nutritional formulary, or laboratory criteria) changed during 2022–2024, and the enhanced nutritional support in the study group was an added intervention component, not a secular trend. However, residual confounding from unmeasured time-varying factors cannot be excluded. The predominance of alcoholic cirrhosis and the short mean abstinence duration before admission imply that continued abstinence during follow-up could have contributed to the observed improvements, making it difficult to completely isolate the specific effect of the nursing intervention. The single-center design may have introduced selection bias, restricting generalizability to other etiologies and settings. Additionally, the modest between-group difference in MELD scores (1.4 points at 12 months) should be interpreted with caution, as its independent clinical significance remains uncertain, though the concurrent improvements in LFI, Child-Pugh distribution, and complication rates support an overall pattern of benefit. Future prospective multicenter studies with prespecified laboratory data collection, extended follow-up, and cost–benefit analysis are warranted to validate and extend our findings.

## Conclusion

In summary, this retrospective study suggests that continuous care based on hierarchical nursing is associated with superior clinical outcomes in patients with liver cirrhosis, including improved liver function, fewer complications, better self-management, and enhanced quality of life. Although prospective validation is needed, this integrated nursing strategy offers a feasible and low-cost approach to optimize long-term cirrhosis care in real-world settings.

## Data Availability

The raw data supporting the conclusions of this article will be made available by the authors, without undue reservation.
